# Vitamin A Emulsion Encapsulated with Whey Protein Isolate–Soybean Lecithin Enhances Surimi Gel Structure and Protein Conformation

**DOI:** 10.3390/foods14203582

**Published:** 2025-10-21

**Authors:** Mulin Chen, Xiaohan Huang, Yawen Lin, Shumin Yi

**Affiliations:** National & Local Joint Engineering Research Center of Storage, Processing and Safety Control Technology for Fresh Agricultural and Aquatic Products, National R&D Branch Center of Surimi and Surimi Products Processing, College of Food Science and Engineering, Bohai University, Jinzhou 121013, China; chenmulin0508@163.com (M.C.); xhclover@163.com (X.H.); linyawen2020@163.com (Y.L.)

**Keywords:** vitamin A, emulsion, gel properties, protein conformation, myofibrillar protein

## Abstract

In this study, whey protein isolate–soybean lecithin-encapsulated vitamin A emulsion (V_A_-WSE) with different oil-to-water ratios was prepared and characterized. The impact of V_A_-WSE on the physicochemical characteristics of *Nemipterus virgatus* surimi at varying concentrations was determined. The V_A_-WSE emulsion exhibited the best stability when the oil-to-water (O:W) ratio was 1:1 (*w*/*w*). Composite gels prepared by mixing V_A_-WSE (O:W = 1:1, *w*/*w*) with surimi at different ratios showed significantly improved gel properties. In particular, the hardness, chewiness, gel strength, and water-holding capacity of the composite gel with a V_A_-WSE concentration of 8% (*w*/*w*) reached the highest values of 2629.00 g, 2051.27 g, 292.16 g·cm, and 87.10%, respectively. Similarly, the observed voids in the microstructural images gradually decreased with rising V_A_-WSE concentration and were the smallest in the 8% sample group. Surimi gels showed remarkably enhanced hydrogen bonds in the V_A_-WSE concentration range of 0–8%, increasing from 0.001 to 0.025 mg/mL (*p* < 0.05). Furthermore, it was observed that the energy storage modulus (G′) was larger than the loss modulus (G″), suggesting the dominant elastic characteristics of the composite gels. The solubility and total sulfhydryl group contents significantly increased from 30.33 to 88.29% and from 4.90 to 28.19 nmol/mg, respectively. In summary, V_A_-WSE can promote the unfolding of the myofibrillar protein (MP) structure and improve the gel properties of surimi gels. These results support the development of functional surimi products.

## 1. Introduction

Surimi is essentially a concentrate of myofibrillar proteins (MPs), prepared through processes such as meat harvesting, rinsing, and fine filtration [1]. Generally, the protein-rich surimi has a mild flavor and is used as an intermediate raw material for the development of various value-added products [2,3]. Unique-textured and nutritionally rich surimi products, such as fish sausages, fish balls, and fish cakes, are increasingly favored by consumers. During surimi processing, the rinsing step plays a crucial role in determining the final product quality. It removes some water-soluble components, enzymes, blood, and other impurities, thereby improving the color and enhancing the gel properties. However, some lipids are lost, which may not only adversely affect the flavor profile of products but also reduce their nutritional value, thereby decreasing consumer acceptability [4]. Studies have been conducted to fortify surimi products by adding exogenous lipids to address issues arising from rinsing [5]. Exogenous lipids improve the gel properties and appearance of surimi through both physical filling and chemical interactions, while also enhancing the release of flavor compounds. Among them, vegetable oils are considered a preferred alternative to animal fats for enhancing the nutritional value of surimi products due to their richness in unsaturated fatty acids [6]. Studies have shown that the addition of an appropriate amount of camellia oil (8 g/100 g) to surimi gel can improve its whiteness, hardness, water-holding capacity (WHC), and overall acceptability. Additionally, camellia oil can fill the gaps in the gel matrix, resulting in a denser gel network structure [5]. The addition of virgin coconut oil reduces the formation of undesirable flavor compounds and enhances the overall sensory quality of surimi gel [7]. Nevertheless, the direct and excessive incorporation of vegetable oils into surimi gels has the potential to modify the configuration of MPs, weakening texture and gel strength [8,9]. The direct addition of soybean oil, peanut oil, and corn oil can improve the whiteness of surimi gel, but it adversely affects the WHC and gel strength of surimi gel while disrupting its three-dimensional network structure [10]. How to add oils to surimi gels without affecting the quality of the product remains a key challenge. In order to solve this problem, researchers have applied emulsification technology to surimi products. Emulsified oil can be filled in the network by copolymers or fillers to form dense gel structures, which play a crucial role in water retention [1,11]. *Litsea cubeba* oil is rich in citral, possessing a strong lemon aroma, which is suitable for improving off-flavors in aquatic products. It also exhibits excellent antioxidant and antibacterial activity [12,13]. Sun et al. applied *Litsea cubeba* oil to surimi and found that, compared to surimi gels with directly added *Litsea cubeba* oil, gels incorporated with casein-stabilized *Litsea cubeba* oil-high internal phase emulsions (LCO-HIPEs) showed higher WHC, whiteness, and a denser gel structure. Additionally, LCO-HIPEs effectively slowed lipid oxidation in surimi gel, preserving higher concentrations of flavor compounds, thereby contributing to the development of surimi products with improved flavor profiles [13]. As a valuable source of n-3 fatty acids, diacylglycerol (DAG) is increasingly being adopted as a fat substitute in the development of functional products [14,15]. However, the poor oxidative stability of DAG typically necessitates its emulsification prior to application in food development [1]. Studies have demonstrated that a moderate concentration of DAG emulsion (5%) can significantly enhance the whiteness, gel strength, and hardness of surimi gels while also enriching the flavor compounds [14]. Additionally, Mi et al. showed that adding a modified cellulose-based emulsion (5%) resulted in higher gel strength and texture compared to surimi gels directly supplemented with flaxseed oil [16].

The aforementioned traditional emulsions, such as *Litsea cubeba* oil, DAG, and cellulose-based systems, have been applied in surimi gels primarily to develop surimi products with desirable flavor and texture through the mechanisms of fat replacement and water retention. Recent studies have focused on encapsulating or loading bioactive compounds such as fat-soluble vitamins, flavonoids, β-carotene, and curcumin based on emulsion systems [17,18]. On the one hand, these bioactive compounds with poor stability and low bioavailability are precisely encapsulated within droplets to prevent degradation caused by oxidation, light exposure, or enzymatic hydrolysis. This enables the controlled release of the active ingredients at targeted sites such as the intestine, thereby enhancing their bioavailability [18]. On the other hand, improvements in food flavor and texture through traditional emulsions can strengthen the nutritional value of foods, providing essential raw materials for the development of functional foods. Vitamin A is an essential fat-soluble vitamin for humans with a wide range of functions. It can maintain the normal metabolism and function of the human body and has positive effects on vision, epithelial surfaces, embryonic growth and development, immune ability, reproduction, etc. [19]. Nevertheless, vitamin A has poor stability and low bioavailability. It is susceptible to chemical degradation under normal storage conditions. Under extreme conditions (including high temperatures, high oxygen, acidity, and light levels), vitamin A may degrade rapidly [20]. Emulsion-based delivery systems are widely used for vitamin A encapsulation. Previous studies have revealed that oil-in-water (O/W) emulsions are the most economical and effective carriers of lipophilic compounds. Furthermore, encapsulating vitamin A in O/W emulsions can make vitamin A more stable and bioavailable [21,22]. Dissolving vitamin A in virgin coconut oil and using isolated whey protein to wrap it in an O/W emulsion could improve its stability and bioavailability [23].

Whey protein isolate (WPI) has efficient emulsifying properties in emulsions. Soy lecithin (SL) has excellent emulsifying capacity based on its amphiphilic molecular structure [24]. However, due to the sensitivity of SL to ionic environments, it is not sufficient to maintain its long-term stability. Therefore, it is often used in association with other emulsifiers and stabilizers [22,25]. In aqueous solutions, non-covalent binding can be achieved between WPI and SL via hydrophobic forces and hydrogen bonds. Their interaction makes curcumin emulsions more stable [22].

Even though extensive studies have been conducted on the use of whey protein isolate–soy lecithin (WPI–SL) as an emulsifier for emulsions, the preparation of WPI–SL-encapsulated bioactive compound emulsions and their application in surimi and surimi products have rarely been investigated. In this study, a WPI–SL-encapsulated vitamin A emulsion (V_A_-WSE) was incorporated into surimi gels. The present study elucidated the impact of V_A_-WSE concentration on *Nemipterus virgatus* surimi in terms of the gel properties and MP conformation. This research contributes to a theoretical foundation and provides technical support for the development of functional surimi products.

## 2. Materials and Methods

### 2.1. Materials

Frozen *Nemipterus virgatus* (Grade AA) surimi was provided by Qingdao Baiteng International Trade Co., Ltd. (Qingdao, China). WPI and SL were provided by Jiangsu Baiye Biotechnology Co., Ltd. (Nanjing, China) and Hebei Desong Biotechnology Co., Ltd. (Baoding, China), respectively. Soybean oil was purchased from Jinzhou Supermarket (Jinzhou, China). Vitamin A was obtained from Wenwei Biotechnology Co., Ltd. (Shangqiu, China). The above experimental materials were food grade, while other chemicals were analytical grade.

### 2.2. Preparation of V_A_-WSE

V_A_-WSE was prepared using the modified approaches of Ma et al. [26] and Yi et al. [27]. Briefly, WPI and SL were mixed at a ratio of 1:5 (*w*/*w*) and dissolved in phosphate-buffered saline (PBS, pH 7.0) to obtain a 20% (*w*/*v*) WPI–SL dispersion. Subsequently, the WPI–SL complex, prepared after 8 h of stirring at 20 °C with a magnetic stirrer (SN-MS-6D, Shangpu Instrument Co., Ltd., Shanghai, China), was treated as the aqueous phase. The oil phase was obtained after mixing vitamin A with soybean oil, in which the mass of vitamin A was 0.1% of the soybean oil. Before mixing the vitamin A, the soybean oil was preheated in a water bath (40 °C, 20 min). The oil phase was mixed with the WPI–SL complex at 5:1, 3:1, 1:1, 1:3, and 1:5 ratios. Finally, the oil–water mixture underwent 2 min of homogenization at 10,000 r/min to obtain the emulsion, which was stored at room temperature (RT), protected from light. In addition, the whey protein isolate–soy lecithin emulsion (without vitamin A in the oil phase) was prepared as described above.

### 2.3. Characterization of V_A_-WSE

#### 2.3.1. Microstructure

After mixing V_A_-WSE (1 mL) with Nile blue (40 µL) dye solution, the microstructure of V_A_-WSE was observed using a confocal laser scanning microscope (CLSM, SP8, Leica Instrument Co., Ltd., Wetzlar, Germany) under a 40× objective. Nile blue was excited at an excitation wavelength of 633 nm.

#### 2.3.2. Particle Size

The particle size of V_A_-WSE was measured using a laser particle-size analyzer (Nano-ZS90, Malvern Instrument Co., Ltd., Malvern, UK), according to the method described by Xu et al. [28] with some modifications.

#### 2.3.3. Vitamin A Encapsulation Efficiency

To obtain the vitamin A standard solution, 20 mg of vitamin A standard was dissolved in anhydrous ethanol, fixed to 10 mL, and then diluted 5 times. With a 600 mL/L ethanol solution as a blank control, standard solutions of 0.4, 0.48, 0.56, 0.64, 0.72, and 0.8 mL of vitamin A were precisely transferred into 10 mL brown volumetric bottles, and the volume of the 600 mL/L ethanol solution was fixed to the scale line and adjusted to zero. An ultraviolet–visible spectrophotometer (UV-2550, Shimadzu Instrument Co., Ltd., Kyoto, Japan) was used to measure the absorbance at 327 nm, with the mass concentration of vitamin A and the light absorption value as the horizontal coordinate and vertical coordinate, respectively. The linear regression equation between the mass concentration of vitamin A and the light absorption value was obtained using the least-squares method. Subsequently, 1 mL of V_A_-WSE from the middle of the bottle was dissolved in 9 mL of 600 mL/L ethanol solution and then centrifuged for 15 min at 3000 rpm [29]. The absorbance of the supernatant was detected at the same wavelength. The standard curve equation for vitamin A–anhydrous ethanol was used (y = 274.56x − 4.3254, R^2^ = 0.983, mg/L) to calculate the vitamin A mass. The encapsulation rate of vitamin A was calculated using Equation (1):

(1) $$\mathrm{Encapsulation}\mathrm{efficiency}\left(\%\right)=M_{A}/M_{0}\times 100$$

where x is the absorbance of vitamin A and y is the mass concentration of vitamin A. Furthermore, M_A_ is the mass of encapsulated vitamin A, and M_0_ is the initial mass of vitamin A supplied to the oil phase; both units are expressed in g.

#### 2.3.4. Emulsion Stability Index (ESI) and Storage Stability

The V_A_-WSE (80 μL) was added to a sodium dodecyl sulfate solution (SDS, 0.1% *w*/*v*, 8 mL). Immediately after mixing, the absorbance values were determined by a UV spectrophotometer at a wavelength of 500 nm. After 10 min of standing, the absorbance values were measured using the same experimental procedure [30]. The ESI for the emulsion was determined using Equation (2):

(2) $$\mathrm{ESI}\left(\%\right)=A_{10}/A_{0}\times 100$$

At 25 °C, V_A_-WSE with different oil-phase fractions was put into glass sample bottles, and their appearance changes were observed on days 0, 3, 5, and 7.

#### 2.3.5. Rheological Behavior

The rheological behavior of V_A_-WSE was measured following Li et al. [30] using a rheometer (Discovery DHR-1, TA Instruments, New Castle, DE, USA). The probe was slowly lowered, and after it was stabilized, the excess sample was then scraped off. In the shear strain scan, the shear rate ranged from 0.1 to 1000 s^−1^. In the frequency scan, the angular frequency ranged from 0 to 150 rad/s. All experimental samples were measured at 25 °C.

### 2.4. Preparation of Composite Gels Containing V_A_-WSE

The surimi thawed at 4 °C was chopped for 2 min in a vacuum chopper (UMC5, Stephan Machinery GmbH, Hameln, Germany) without adding any substances. Then, the resulting surimi was mixed with 2.5% (*w*/*w*) NaCl for another 2 min of chopping. Subsequently, V_A_-WSE at varying doses was added to five equal portions of surimi and further chopped for 3 min, and the concentrations of V_A_-WSE in the obtained surimi pastes were 2%, 4%, 6%, 8%, and 10%, respectively. The surimi pastes were vacuumed, sealed in 32 mm folded plastic casings, and heated in two stages (40 °C/30 min, 90 °C/20 min). Finally, the prepared composite gels were placed in ice water to cool to RT and were maintained at 4 °C.

### 2.5. Gel Properties

#### 2.5.1. Gel Strength and Texture Profile Analysis (TPA)

After 30 min of balance at RT, the composite gels were cut into 20 mm high cylinders. Their gel strength was examined using a P/5S spherical plunger probe, and TPA was performed using a P/50 cylindrical probe with a texture analyzer (TA-XT Plus, Stable Micro System, Godalming, UK).

#### 2.5.2. Water-Holding Capacity (WHC) and Cooking Loss (CL)

The samples were sliced and weighed accurately as M_1_ (about 3 g), then wrapped in two layers of filter paper. Following 10 min of centrifugation at 10,000 r/min, the gel samples were removed and weighed once more (M_2_). The WHC was obtained using Equation (3):

(3) $$\mathrm{WHC}\left(\%\right)=M_{2}/M_{1}\times 100$$

The gel samples were weighed (G_1_) after being sliced into cylinders that measured roughly 15 × 15 × 5 mm. Then they were packed into cooking bags, sealed, steamed in a water bath (90 °C, 20 min), and stored in a refrigerator (4 °C, 24 h). After that, the liquid on the surface of the composite gels was absorbed using filter paper and weighed again (G_2_). The CL was calculated using Equation (4):

(4) $$\mathrm{CL}\;(\%)={(G_{1}-G}_{2})/G_{1}\times 100$$

#### 2.5.3. Magnetic Resonance Imaging (MRI)

Following the method of Zhang et al. [31], hydrogen proton density imaging of the composite gels was performed using an MRI analyzer (MesoMR23-040H-I, Suzhou Niumag Co., Ltd., Suzhou, China). The instrument parameters were as follows: a resonance frequency of 21.3 MHz, a magnet temperature of 32 °C, a coil diameter of 60 mm, and a magnetic field strength of 0.55 T.

#### 2.5.4. Whiteness

A color difference meter (CR-400, Konica Minolta, Tokyo, Japan) was employed to detect the L* (brightness), a* (redness/greenness), and b* (yellowness/blueness) values of the samples. The whiteness was calculated using Equation (5):

(5) $$\mathrm{Whiteness}=100-\sqrt{{\left(100-L*\right)}^{2}+{\left(a*\right)}^{2}+{\left(b*\right)}^{2}}$$

#### 2.5.5. Optical Microscopy

A CM-1850 freezing microtome (CM-1850, Leica Instrument Co., Ltd., Wetzlar, Germany) was adopted to observe the microstructure of 10 μm slices of the samples, following Lv et al. [1].

#### 2.5.6. Chemical Forces

The surimi gels (about 2 g) were mixed with 0.05 M NaCl (SA), 0.6 M NaCl (SB), 0.6 M NaCl + 1.5 M urea (SC), and 0.6 M NaCl + 8 M urea (SD), respectively, following the description of Mi et al. [32]. Subsequently, the obtained solutions were sequentially homogenized (16,000 r/min, 15 s) and allowed to stand (4 °C, 1 h), followed by 15 min of centrifugation (10,000 r/min). The hydrogen bonds, ionic bonds, and hydrophobic interactions were determined based on c(SB)-c(SA), c(SC)-c(SB), and c(SD)-c(SC), respectively.

#### 2.5.7. Rheological Behavior

The Peltier temperature control system was adopted here. The samples were heated from 20 to 90 °C at 2 °C/min, set to a frequency of 1 Hz and a stress of 1 Pa to record the storage modulus (G′) and loss modulus (G″) [14].

### 2.6. Extraction of MPs

Following Yi et al. [33], MPs were extracted from the surimi. Briefly, three centrifugations (9000× *g*, 10 min, 4 °C) were performed. The mixture of low-temperature and low-salt PBS (containing 0.02 M Na_2_HPO_4_, 0.02 M NaH_2_PO_4_, and 0.05 M NaCl mixture, pH 7.5) and surimi at a ratio of 4:1 (*w*/*w*) was homogenized under a high-speed homogenizer (T25, IKA, Staufen, Germany) and left for 15 min at 4 °C. Then, a refrigerated centrifuge (Soravall LYNX4000, Thermo, Waltham, MA, USA) was used to centrifuge the mixture to obtain the precipitates. Subsequently, the precipitates were mixed with low-temperature and high-salt PBS (0.02 M Na_2_HPO4, 0.02 M NaH_2_PO4, and 0.45 M NaCl mixture, pH 7.5) at a 1:4 (*w*/*w*) ratio and then stirred (4 °C, 2 h) and centrifuged. After that, after 10-fold dilution in ice water, the diluted supernatant was left for 30 min and then centrifuged. The final precipitates obtained were the MPs.

### 2.7. Preparation of MP Solutions

The MP sol (90 mg/mL) was mixed with V_A_-WSE to undergo homogeneous mixing. The final concentrations of V_A_-WSE in the MP solutions were 2.5%, 5%, 7.5%, and 10% (*w*/*w*). The MP solution without V_A_-WSE was used as the control group.

### 2.8. MP Conformation

#### 2.8.1. Particle Size and Solubility

The particle size of the MP solutions was measured using the equipment referred to in Section 2.3.2. The MP solutions were diluted to 2 mg/mL using 20 mM PBS (0.6 M NaCl, pH 6.0). Subsequently, after 10 min of centrifugation at 5000× *g,* the supernatant was collected. The ratio of protein content in the supernatant to that in the original solution was defined as the solubility of the MPs.

#### 2.8.2. UV Absorption Spectra

The method of Yi et al. [33] was adopted to adjust the mass concentrations of the MP solutions to 0.25 mg/mL, and the UV spectrum was scanned, with PBS (containing NaCl) as the control.

#### 2.8.3. Total Sulfhydryl Group Content

The total sulfhydryl group content was determined following the description of Wu et al. [34]. In brief, 0.5 mL of the MP solutions (1 mg/mL) was mixed with 4.5 mL of Tris–glycine buffer (0.086 M Tris, 8 M urea, 4 mM EDTA, and 0.09 M glycine, pH 8.0). Then, the mixture was reacted with Ellman reagent (0.5 mL) for half an hour at 30 °C. The absorbance was measured at a wavelength of 412 nm.

### 2.9. Statistical Analysis

All experiments were conducted in triplicate unless otherwise noted. Data analysis was performed using SPSS 26.0 software (IBM Corp., Armonk, NY, USA). Charts were plotted using Origin 2022 (OriginLab Corporation, Northampton, MA, USA) and GraphPad Prism 10.1.2 (GraphPad Software, Inc., San Diego, CA, USA). Statistical analysis was performed using one-way ANOVA and two-factor correlation analysis; *p* < 0.05 represented statistical significance.

## 3. Results and Discussion

### 3.1. V_A_-WSE Characterization

#### 3.1.1. Microstructure

**Figure 1:**
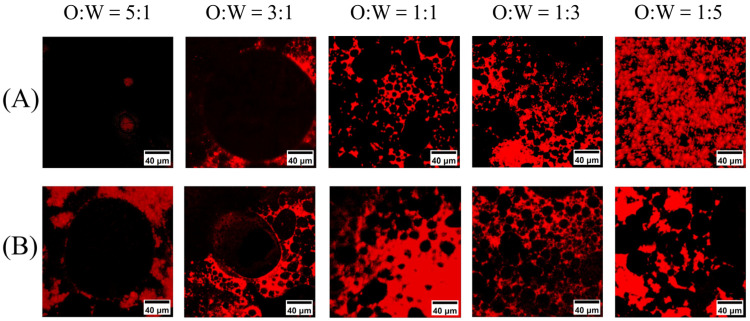
CLSM images of WSE (**A**) and V_A_-WSE (**B**) with different oil–water ratios. WSE: whey protein isolate–soy lecithin emulsion; V_A_-WSE: whey protein isolate–soybean lecithin-encapsulated vitamin A emulsion. The oil–water ratios of the prepared WSE and V_A_-WSE were 5:1, 3:1, 1:1, 1:3, and 1:5, respectively.

The microstructures of the WPI–SL emulsion (WSE) and V_A_-WSE with varying oil–water ratios are shown in Figure 1A,B. Red areas indicate proteins, while black areas represent oil droplets. It was also demonstrated that WSE and V_A_-WSE prepared in the experiment were typical O/W emulsions. As shown in Figure 1B, at oil–water ratios of 1:1 and 1:3, the oil droplets squeezed and deformed each other, thereby making the structure of V_A_-WSE more stable. CLSM images showed that for both WSE and V_A_-WSE, the emulsion droplets in the 5:1 and 3:1 oil–water ratio groups were large and unevenly distributed. Moreover, the phenomenon of droplet fusion in WSE was observed (Figure 1A). High-internal-phase Pickering emulsions stabilized by SMP exhibited fusion at oil-phase volume fractions of 70–90% [35]. Compared to WSE, the large droplets of V_A_-WSE were fewer in number, and the droplet distribution was uniform, with no obvious aggregation trend. This might be because V_A_-WSE formed a steady network that preserved the rigid structures between the emulsion droplets. As a result, full contact and fusion were hardly achieved among the droplets [36].

#### 3.1.2. Particle Size

**Figure 2:**
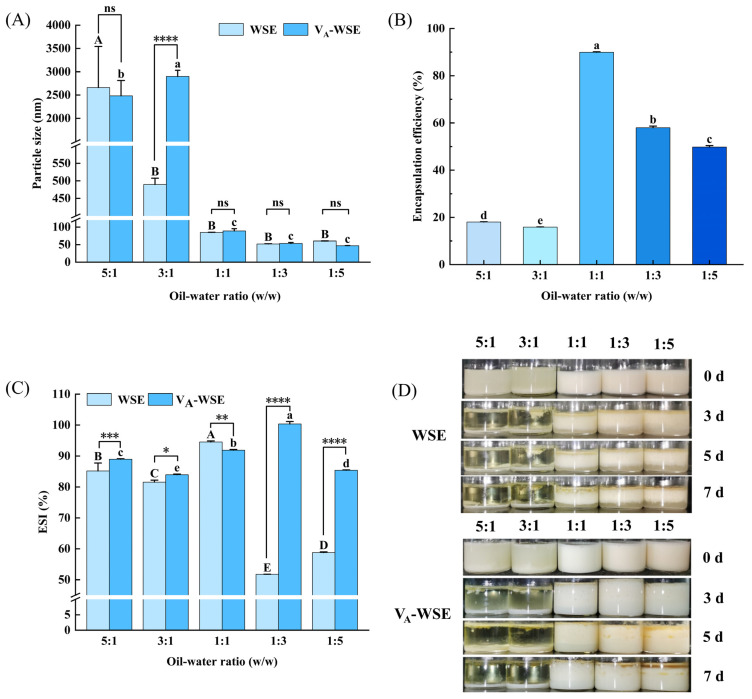
Changes in particle size (**A**), vitamin A encapsulation efficiency (**B**), ESI (**C**), and storage stability (**D**). WSE: whey protein isolate–soy lecithin emulsion; V_A_-WSE: whey protein isolate–soybean lecithin-encapsulated vitamin A emulsion. The oil–water ratios of the prepared WSE and V_A_-WSE were 5:1, 3:1, 1:1, 1:3, and 1:5, respectively. Note: ns, *, **, ***, and **** indicate non-significant, *p* < 0.05, *p* < 0.01, *p* < 0.001, and *p* < 0.0001, respectively, between different emulsion groups at the same oil–water ratio. Different letters (a–e, A–E) in the same column indicate significant differences at *p* < 0.05.

Emulsion droplet size is a pivotal metric for assessing emulsion stability. As illustrated in Figure 2A, the particle size of V_A_-WSE (46.96–88.92 nm) did not show any obvious differences (*p* > 0.05) when the oil–water ratios were less than or equal to 1. However, the particle size (2483.93–2901.27 nm) increased significantly when the oil–water ratios were 5:1 and 3:1 (*p* < 0.05). As the oil-phase contents in V_A_-WSE increased, the WPI content was insufficient to completely cover the oil-droplet surface, causing them to flocculate, which increased particle size [37]. Additionally, the particle sizes of WSE and V_A_-WSE with the same oil–water ratio (1:5 to 1:1) showed no obvious difference. This might be due to the small amount of vitamin A added in the oil phase, which had little effect on the emulsion particle size. The emulsion prepared by Banasaz et al. [21] with a pressure of 100 MPa had the smallest particle size and uniform distribution, and the addition of vitamin A did not significantly change this result.

#### 3.1.3. Vitamin A Encapsulation Efficiency

Emulsion systems loaded with vitamin A were prepared with different oil–water ratios using WPI–SL complex as an emulsifier and vitamin A as a fat-soluble bioactive substance, and the loaded delivery capacity of this emulsion system for vitamin A was evaluated based on the encapsulation rate. As illustrated in Figure 2B, the encapsulation rate of vitamin A was 89.91% for samples with an oil–water ratio of 1:1, which was observably improved (*p* < 0.05). At this oil-to-water ratio, the emulsion formed a thicker and more compact interfacial protein film, thereby enhancing the stability of the V_A_-WSE and effectively encapsulating vitamin A to minimize depletion due to degradation from exposure. However, the encapsulation rate of vitamin A was under 20% at oil–water ratios of 5:1 and 3:1. This phenomenon occurred because at high oil-to-water ratios, oil-droplet aggregation reduced emulsion stability, thereby decreasing the encapsulation efficiency [38].

#### 3.1.4. ESI and Storage Stability

The ESI represents the ability of an emulsifier to maintain the stability of the emulsion and is an essential indicator for evaluating emulsification performance. As shown in Figure 2C, the ESI of the V_A_-WSE was markedly higher than that of other oil–water ratios when the oil–water ratios were 1:1 and 1:3 (*p* < 0.05). In the range of oil–water ratios from 1:5 to 1:1, this might be because the interfacial area increased with the rise in the oil–water ratio, resulting in more protein adsorption sites, which led to greater interfacial adsorbed protein content and ultimately to the formation of protein-stabilized oil droplets, thereby increasing the ESI [8]. An appropriate oil–water ratio made the emulsifier complex sufficient to cover the oil–water interface, thereby strengthening the emulsion stability. Furthermore, the ESI of V_A_-WSE was significantly different from that of WSE for the same oil-to-water ratio (*p* < 0.05). This phenomenon occurred because vitamin A is a fat-soluble substance that reduces surface tension at the oil–water interface, thus contributing to a more stable emulsifying system [39]. Meanwhile, the WPI–SL complex could form a stable interfacial film; therefore, the addition of vitamin A might have further strengthened the stability of the interface film, making V_A_-WSE more stable [22].

The appearance of WSE and V_A_-WSE was observed after storage at RT for 0, 3, 5, and 7 days (Figure 2D). After standing in storage for 3 days, significant phase separation was observed in emulsions with oil–water ratios of 5:1 and 3:1. The oil-phase mass fraction was too large, breaking the balance of the oil–water interfacial tension, making WSE and V_A_-WSE lose stability, and resulting in phase separation. The reduction in the aqueous phase hindered the WPI–SL complex from stabilizing excess oil droplets, which resulted in phenomena such as agglomeration and flocculation of the emulsions and diminished the storage stability of the emulsions [40]. At an oil–water ratio of less than or equal to 1, WSE displayed oil precipitation on the third day of storage. Compared with WSE, V_A_-WSE was more stable and less prone to delamination. This phenomenon was due to the hydrophobicity of vitamin A, which made the WPI–SL more easily adsorbed onto the surface of the liquid drops, increasing the charge of oil droplets and more deeply stabilizing the emulsion’s spatial structure [39,41]. V_A_-WSE with a 1:1 oil–water ratio had the highest stability, and the vitamin A encapsulation efficiency shown in Figure 2B supports this result.

#### 3.1.5. Rheological Behavior

**Figure 3:**
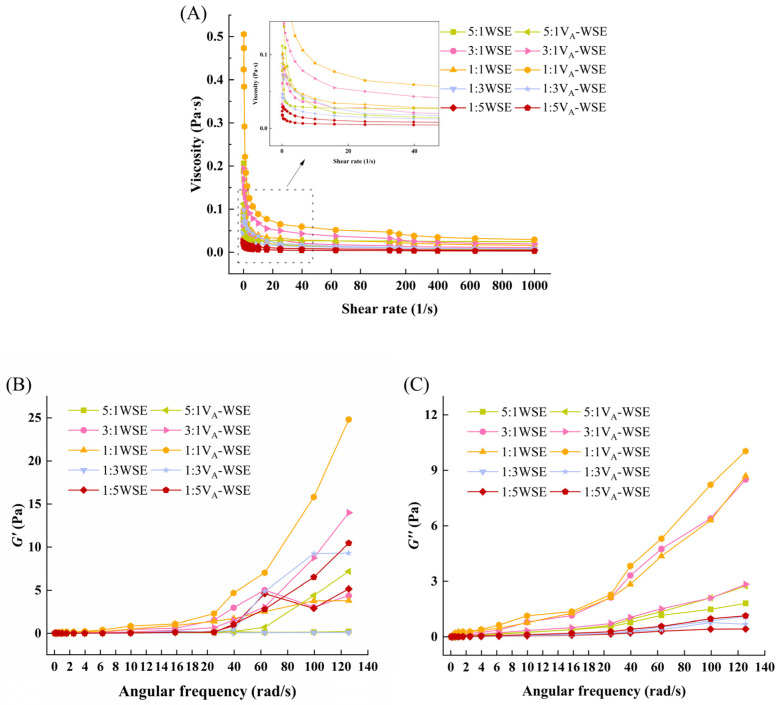
Changes in apparent viscosity (**A**), G′ (**B**), and G″ (**C**) of WSE and V_A_-WSE. WSE: whey protein isolate–soy lecithin emulsion; V_A_-WSE: whey protein isolate–soybean lecithin-encapsulated vitamin A emulsion. The oil–water ratios of the prepared WSE and V_A_-WSE were 5:1, 3:1, 1:1, 1:3, and 1:5, respectively.

The apparent viscosity changes in WSE and V_A_-WSE, along with the shear rate, are depicted in Figure 3A. Both emulsions exhibited lower apparent viscosity as the shear rate increased, revealing that they were typical non-Newtonian pseudoplastic fluids with time-dependent shear-thinning behavior, which was independent of the addition of vitamin A. To put it another way, the fluidity of WSE and V_A_-WSE improved at higher shear rates. This was because the internal structures of the emulsions were damaged and reorganized at rising shear rate, resulting in reduced flow resistance [42]. Compared with WSE, V_A_-WSE had higher viscosity, and the initial viscosity was the highest when the oil–water ratio was 1:1, indicating that it had strong network stability.

Figure 3B,C illustrate the angular frequency-dependent changes in the G′ and G″ values. From a rheological point of view, V_A_-WSE can be classified as a typical weak gel. V_A_-WSE showed a higher G′ value than WSE, which indicated that it had stronger elastic characteristics. Under the action of the WPI–SL and vitamin A, the emulsion droplets were tightly wound and formed a dense network, which limited their mobility and ultimately improved the G′ value of the V_A_-WSE [43]. In this work, the observed G″ value had a similar trend to the G′ value. The V_A_-WSE with a 1:1 oil–water ratio had the highest G′ and G″ values, and the frequency dependence was weak, indicating the strongest network structure.

### 3.2. Impact of Changes in V_A_-WSE on Gel Properties

#### 3.2.1. Gel Strength and TPA

**Figure 4:**
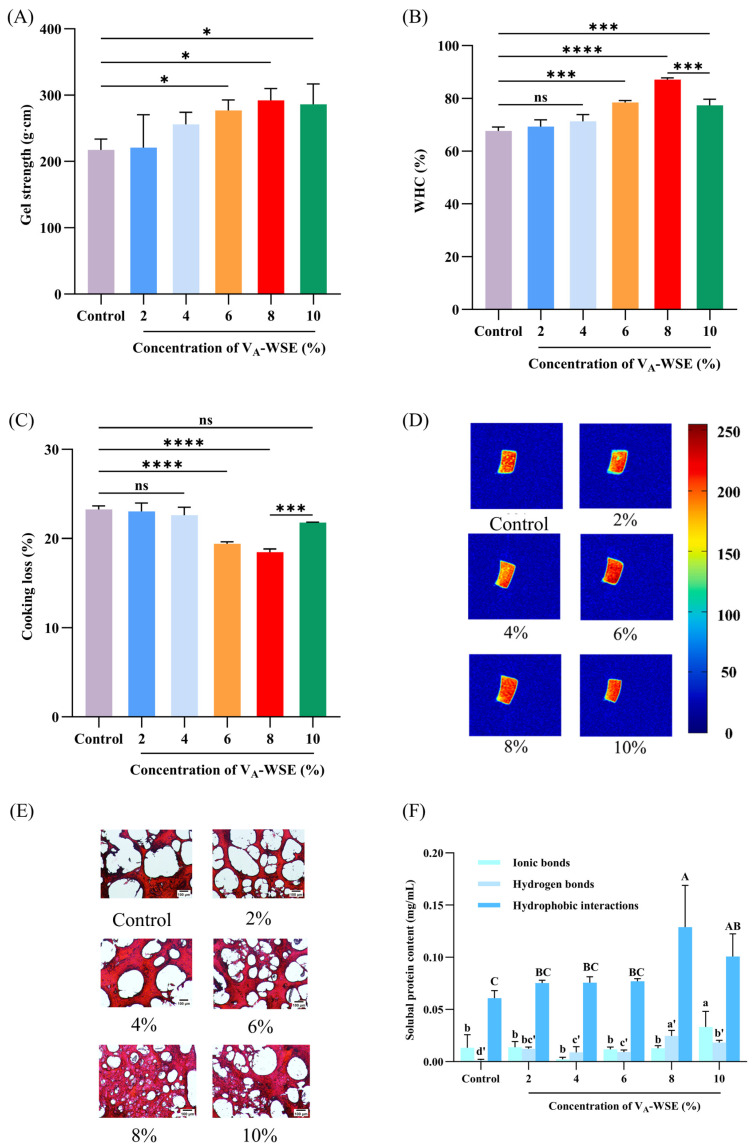
Changes in gel strength (**A**), WHC (**B**), CL (**C**), MRI (**D**), microstructure (**E**), and chemical forces (**F**) of surimi gels with different concentrations of V_A_-WSE. V_A_-WSE: whey protein isolate–soybean lecithin-encapsulated vitamin A emulsion. The concentrations of V_A_-WSE in the prepared surimi gels were 0% (control), 2%, 4%, 6%, 8%, and 10%, respectively. Note: ns, *, ***, and **** indicate non-significant, *p* < 0.05, *p* < 0.001, and *p* < 0.0001, respectively. Different letters (a,b, a’–d’, A–C) in the same column indicate significant differences at *p* < 0.05.

The capability of surimi to gel when heated is reflected in the gel strength, which is a combination of gel elasticity and hardness. When the concentrations of V_A_-WSE were 2%, 4%, 6%, 8%, and 10%, respectively, the gel strength of the composite gels improved by 1.5%, 17.6%, 27.2%, 34.3%, and 31.6% in comparison to the pure surimi gel (Figure 4A). The gel strength demonstrated a maximum value of 292.16 g·cm when the emulsion concentration was 8%. At this point, the emulsion could fully fill the gel system in the form of small fat globules, which played a supporting and reinforcing role and contributed to the formation of the protein gel network [3]. However, adding 10% V_A_-WSE slightly reduced the gel strength compared to 8% V_A_-WSE. Similar results were obtained by Mi et al. [32], where oil droplets restricted the cross-linking between proteins upon the addition of excessive emulsion, thereby reducing the gel strength of surimi gels.

TPA can be used to characterize the structural properties of surimi gels. As shown in Table 1, the addition of V_A_-WSE improved the TPA of the surimi gels to varying degrees. The gel hardness showed the same variation trend as the gel strength (Figure 4A). Compared to the pure gel (1144.43 g), the hardness of the surimi gels containing the appropriate concentration of V_A_-WSE (2–8%) increased from 1355.03 to 2629.00 g. Smaller emulsified droplets exhibited an even distribution throughout the protein network structures, which resulted in a higher resistance to external forces, thereby effectively enhancing the hardness of surimi gels [44]. Mi et al. reported similar results, showing that the hardness of the surimi gels incorporating appropriate amounts of modified cellulose-based emulsions (2.5–5%) increased with increasing emulsion concentration [16]. Similarly, when DAG emulsions (1–7%) were incorporated into surimi, the gel hardness increased with increasing concentration [14]. Nevertheless, when the concentration of V_A_-WSE was greater than 8%, the hardness, gumminess, and chewiness decreased, likely due to the limited ability of protein to encapsulate oil droplets, thereby marginally decreasing the TPA [14].

#### 3.2.2. WHC and CL

A dense and ordered gel network limits water molecules’ mobility and reduces water loss, thereby increasing the WHC [16]. Remarkably, the V_A_-WSE modified the WHC and CL of the surimi gels, and the amount of emulsion exerted a particularly significant impact (Figure 4B,C). In the range of V_A_-WSE concentrations (2–4%), there were no remarkable changes in the water retention and water loss of the composite gels compared to the pure gel. As the concentration of V_A_-WSE increased, the composite gel in the 8% group had the highest WHC (87.10%) and the lowest CL (18.50%). However, the samples showed an obvious decrease in the WHC (77.35%) at concentrations > 8% (*p* < 0.05). The capacity for exceptional water retention is frequently associated with the presence of dense and regular gel networks [1]. The oil droplets encapsulated by the emulsifier WPI–SL complex participated as copolymers in forming the composite gels, promoting intermolecular cross-linking. The denser 3D network structure benefited the capture of a larger amount of free water. In addition, an appropriate amount of V_A_-WSE enhanced the interaction of the emulsion droplets with surimi proteins, promoting a denser gel network and further preventing water loss [1]. However, excessive filling with V_A_-WSE could lead to the network structure being destroyed and negatively affect the water retention capacity.

#### 3.2.3. MRI

As a fast and non-destructive method, MRI is considered to complement low-frequency magnetic resonance, which can visually monitor water migration in gels on pseudo-color maps based on the depth of gel color, thus determining the distribution of water in food and visualizing internal structural changes [45]. Red and blue indicate higher and lower densities of hydrogen protons in a gel, respectively. Hydrogen proton-weighted images of surimi gels containing different emulsion contents are shown in Figure 4D. As the concentration of V_A_-WSE increased, the hydrogen proton density in the surimi gels increased and then decreased, with the 7.5% sample group exhibiting the brightest red color, indicating that more water was retained inside the gel. This result was consistent with the WHC and CL (Figure 4B,C). This phenomenon may be attributed to the emulsion’s promotion of myofibrillar protein unfolding, leading to the exposure of internal hydrophobic sulfhydryl groups, which promoted the formation of a more stable three-dimensional network structure and enhanced protein–water binding capacity [14].

#### 3.2.4. Whiteness

Whiteness is a significant sensory factor in determining the surimi product quality, exerting a direct impact on consumer acceptance. There is a close association between whiteness changes and protein composition in gels, the denaturation degree, polymerization and cross-linking, and the optical properties of their surfaces. The whiteness values of the gel samples increased and then decreased (Table 2). The whiteness was not remarkably different between the composite gels (containing 2–4% V_A_-WSE) and the control group (76.64). The whiteness and L* values of the composite gels rose markedly (*p* < 0.05) at >4% V_A_-WSE concentration and reached a maximum value (80.78) in the 8% group. The soybean oil in V_A_-WSE penetrated into the surimi gel and changed its internal structure by influencing the interactions between protein molecules. The emulsified oil droplets interacted with the MPs, strengthening the intermolecular cross-links and leading to more optical reflections, thereby elevating the whiteness and brightness values [46]. However, excessive addition of V_A_-WSE (>8%) resulted in a lower whiteness value. This might be because the color of the soybean oil and vitamin A affected the appearance of the composite gels [11]. In fact, as shown in Figure 2D, the prepared V_A_-WSE had a yellowish appearance.

### 3.3. Impact of Changes in V_A_-WSE on Microstructure of Surimi Gels

As displayed in Figure 4E, the pure gel (without the addition of V_A_-WSE) showed a loose, unevenly distributed network with large pores. The composite gel networks in the 2% and 4% groups had densities comparable to the pure gel. As the concentration of V_A_-WSE increased (6–8%), a denser and stronger gel network structure was established, and the voids became narrower. Collectively, the gel network structure could be more remarkably enhanced by increasing the concentration of V_A_-WSE in the range of 2–8%, which corresponded to the improved gel strength and WHC (Figure 4A,B). Cross-linking and aggregation between surimi protein molecules were enhanced by appropriate amounts of V_A_-WSE filled into the gels [14]. However, when V_A_-WSE was added at a level greater than 8%, there was a tendency for the pore space of the gel network to expand. This might be explained by the cross-linking and aggregation interference of V_A_-WSE on surimi proteins that was progressively significant at high concentrations, along with a weakened cross-linking degree between the proteins, which ultimately weakened the gel network in the 10% group [47]. Furthermore, an excess of soybean oil was incorporated into the gel samples, resulting in the aggregation of oil droplets. This phenomenon enlarged the protein molecular distance, thereby preventing protein network structures from being formed [48].

### 3.4. Impact of Changes in V_A_-WSE on Chemical Forces of Surimi Gels

Figure 4F illustrates the impact of varying V_A_-WSE concentrations on the intermolecular forces (hydrophobic interactions, hydrogen bonds, and ionic bonds) of the composite gels. The analysis revealed that hydrophobic interactions constituted a greater percentage of the composite gels, while the hydrogen and ionic bonds accounted for a lower proportion, suggesting that the hydrophobic interactions were the primary force in the composite gels. The surimi gels (containing V_A_-WSE) exhibited a dramatic increase in hydrogen bonds compared to the pure gel (*p* < 0.05). The interfacial protein membrane effectively encapsulated trace-emulsified oil droplets and considerably increased the system’s specific surface area, thereby facilitating the formation of hydrogen bonds by enhancing protein intermolecular interactions [16]. However, the gel exhibited reduced hydrophobic interactions and hydrogen bonds when the V_A_-WSE concentration was greater than 8% (*p* < 0.05). Excessive oil droplets hindered protein re-arrangement and unfolding and detrimentally affected protein hydration, thereby reducing hydrophobic interactions in the surimi gels [49]. The proteins in V_A_-WSE competed with the MPs for water molecules, which caused the MPs to interact more weakly with water molecules, resulting in reduced hydrogen bonds [50].

### 3.5. Impact of Changes in V_A_-WSE on Rheological Behavior of Surimi Gels

**Figure 5:**
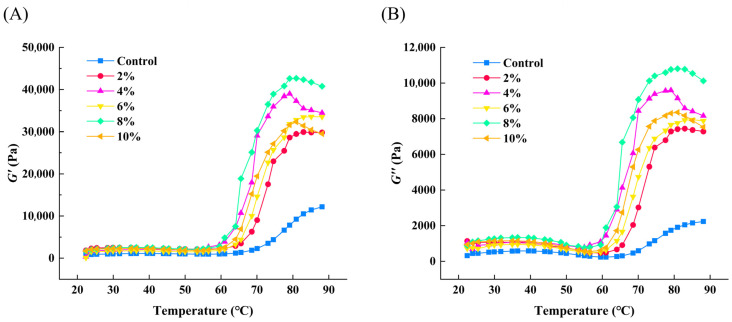
Changes in G′ (**A**) and G″ (**B**) of surimi gels with different concentrations of V_A_-WSE under temperature scanning. V_A_-WSE: whey protein isolate–soybean lecithin-encapsulated vitamin A emulsion. The concentrations of V_A_-WSE in the prepared surimi gels were 0% (control), 2%, 4%, 6%, 8%, and 10%, respectively.

At 20–90 °C, the G′ and G″ values of different gel samples exhibited similar trends (Figure 5). As the temperature rose during the initial heating phase, the G′ and G″ values rose as well, peaking at about 35 °C. At this time, part of the actomyosin aggregated and formed an attenuated gel network through the hydrogen bonds [51]. Then, the G′ and G″ values decreased with increasing temperature, reaching a minimum at around 55 °C. The G′ and G″ values were reduced, possibly due to the broken hydrogen bonds and dissociated protein aggregation, as well as endogenous proteolytic enzyme-induced protein degradation [14]. Upon further heating, the G′ and G″ values rose again and reached a second peak at about 80 °C, accompanied by the formation of stable gel structures by actomyosin. In this temperature range, actomyosin unfolded when exposed to active groups (sulfhydryl and hydrophobic groups); hence, a dense elastic gel network structure was formed [52].

The G′ value reflects the gel’s elastic properties, and a larger G′ value indicates an enhanced gel network structure. At rising temperatures, the composite gels presented significantly increased G′ values compared to the control group. Adding an emulsion containing soybean oil to MP sols could enhance the gel structure [11]. Additionally, the fat globules in V_A_-WSE interacted with the proteins, thereby filling the voids in the gel network and forming a more compact structure, which resulted in a larger G′ value [14]. The surimi gel with 8% V_A_-WSE had the highest G′ value. The G′ value was higher than the G″ value throughout the heating phase, indicating that elastic behavior predominated.

### 3.6. Impact of Changes in V_A_-WSE on MP Conformation

#### 3.6.1. Particle Size and Solubility

**Figure 6:**
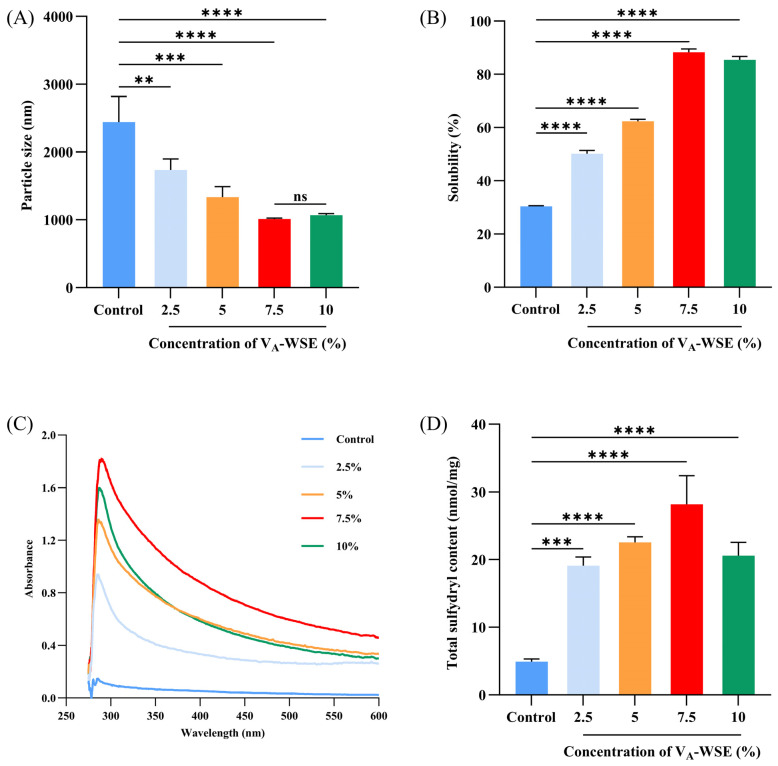
Changes in particle size (**A**), solubility (**B**), UV absorption spectra (**C**), and total sulfhydryl group content (**D**) of MPs with different concentrations of V_A_-WSE. V_A_-WSE: whey protein isolate–soybean lecithin-encapsulated vitamin A emulsion. The concentrations of V_A_-WSE in the prepared MP solutions were 0% (control), 2.5%, 5%, 7.5%, and 10%, respectively. Note: ns, **, ***, and **** indicate non-significant, *p* < 0.01, *p* < 0.001, and *p* < 0.0001, respectively.

The particle size represents the spatial structural changes of MPs, which increase as the protein is denatured and aggregated. As depicted in Figure 6A, V_A_-WSE-added MPs had a markedly reduced particle size (*p* < 0.05) compared to the control (2440.95 nm). With increasing concentrations of V_A_-WSE (0–7.5%), the particle size decreased from 2440.95 to 1012.54 nm. The particle size of the MPs did not show a substantial difference (*p* > 0.05) between the 7.5% and 10% V_A_-WSE groups. V_A_-WSE facilitated the structural unfolding of the MPs, which led to a more homogeneous dispersion in the system, thus reducing the aggregation of MP particles [53]. In addition, the WPI–SL complex in V_A_-WSE may adsorb onto the surface of MPs, increasing the number of negatively charged groups and thereby strengthening the electrostatic repulsion among MPs. In general, the particle size of MPs decreases with increasing electrostatic repulsion [54]. However, the infiltration of excess soybean oil might disrupt the conformation of MPs, exposing more hydrophobic groups that bond with each other through hydrophobic interactions; as a result, the MPs aggregate into larger particles [11].

Protein solubility reflects the protein degradation degree, and decreased solubility marks protein denaturation. The solubility of the MPs rose significantly with increasing V_A_-WSE concentration, as illustrated in Figure 6B (*p* < 0.05), and peaked at 88.29% at a VA-WSE concentration of 7.5%, which was 57.96% higher than that of the control (30.33%). The emulsifier WPI–SL complex was adsorbed onto the MPs’ surface, resulting in a larger number of hydrophilic groups on the surface. This increased the spatial resistance between the MP molecules, reduced their interaction and aggregation, and increased the affinity between the MPs and water molecules, thereby improving solubility [53]. In addition, the magnitude of solubility was affected by changes in protein conformation. As shown in Figure 6A, which illustrates the particle size of the MPs, adding V_A_-WSE reduced the particle size, and a smaller particle size would increase the protein–water molecule interaction, leading to stronger solubility.

#### 3.6.2. UV Absorption Spectroscopy

UV absorption spectra can characterize protein tertiary structures, and most proteins have absorption peaks near 280 nm, which depend on the aromatic amino acids (tyrosine, tryptophan, and phenylalanine) in the protein’s chemical structure [33]. The main chromophore within 250–260 nm is the phenylalanine residue, and the major chromophores at 260–290 nm and near 292 nm are tyrosine and tryptophan, respectively. As displayed in Figure 6C, the characteristic peaks of the MPs for each group appeared at about 275 nm, which was attributed to the pronounced electronic transitions of tyrosine on the peptide chains. V_A_-WSE significantly increased the absorbance values of MP solutions (*p* < 0.05). This might be because V_A_-WSE interacting with the MP solutions changed the microenvironment in which the protein molecules were located, causing a conformational change in the MPs and exposing more chromophore groups [33]. The peak value was the highest in the MP solution containing 7.5% V_A_-WSE, indicating greater structural unfolding of the protein and more chromophore groups exposed to the microenvironment. However, the decrease in the peak at 10% might be explained by the formation of a high-molecular-weight polymer, which caused some non-polar aromatic amino acid residues to be partially masked.

#### 3.6.3. Total Sulfhydryl Group Content

MP is a complex protein, including myosin, actin, tropomyosin, etc. The total sulfhydryl group includes the active sulfhydryl group and the hidden sulfhydryl group, with the former located in the head of myosin and readily oxidized to form a disulfide bond [55]. As shown in Figure 6D, the total sulfhydryl groups of the MP solutions exhibited an obvious increase (*p* < 0.05) as V_A_-WSE concentrations increased, peaking at 28.19 nmol/mg when the V_A_-WSE concentration was 7.5%. Based on the above descriptions, the increase in the total sulfhydryl groups can be accounted for by changes in protein conformation. The formation of smaller MP particle sizes exposed more active and hidden sulfhydryl groups, as reported by Zhang et al. [56]. The observed particle size results also support the changes in the total sulfhydryl groups (Figure 6A). Nevertheless, when V_A_-WSE was further increased to 10%, the total sulfhydryl groups decreased from 28.19 to 20.59 nmol/mg. This might be because the vitamin A in the emulsion promoted oxidation, and the sulfhydryl groups were oxidized to disulfide bonds [55,56]. Moreover, when V_A_-WSE is excessive, the oxidation effect may be more significant.

## 4. Conclusions

V_A_-WSE was prepared and applied to *Nemipterus virgatus* surimi to analyze the changes of various concentrations of V_A_-WSE on the physicochemical behaviors of gel and MP. In this study, V_A_-WSE with an oil–water ratio of 1:1 exhibited the best stability. Additionally, the concentration of V_A_-WSE (8%) significantly (*p* < 0.05) enhanced the gel strength, hardness, and WHC of surimi gels, as supported by a denser and more uniform microstructure with smaller voids. Furthermore, the ultraviolet absorption spectroscopy further revealed that V_A_-WSE interacted with MPs, inducing conformational transitions that improved MP stability and promoted the formation of a more ordered gel network. Nevertheless, excessive addition of V_A_-WSE caused adverse effects. In summary, V_A_-WSE enhances surimi gel properties and supports functional product development.

## Figures and Tables

**Table 1:** Changes in TPA of surimi gels with different concentrations of V_A_-WSE.

V_A_-WSE Concentration (%)	Hardness (g)	Springiness	Gumminess (g)	Chewiness (g)
Control	1144.43 ± 74.00 ^e^	0.98 ± 0.01 ^a^	1025.95 ± 112.83 ^d^	1005.76 ± 119.10 ^d^
2	1355.03 ± 109.44 ^e^	0.95 ± 0.03 ^abc^	1185.75 ± 84.51 ^d^	1131.46 ± 99.29 ^d^
4	1727.32 ± 175.68 ^d^	0.96 ± 0.01 ^ab^	1457.60 ± 127.36 ^c^	1401.60 ± 109.60 ^c^
6	2117.15 ± 49.450 ^c^	0.95 ± 0.01 ^abc^	1783.39 ± 41.53 ^b^	1695.70 ± 58.39 ^b^
8	2629.00 ± 68.61 ^a^	0.94 ± 0.01 ^bc^	2181.73 ± 56.89 ^a^	2051.27 ± 70.61 ^a^
10	2382.51 ± 133.33 ^b^	0.92 ± 0.03 ^c^	1993.61 ± 129.63 ^a^	1842.44 ± 179.42 ^ab^

V_A_-WSE: whey protein isolate–soybean lecithin-encapsulated vitamin A emulsion. The concentrations of V_A_-WSE in the prepared surimi gels were 0% (control), 2%, 4%, 6%, 8%, and 10%, respectively. Different lowercase letters (a–e) in the same column indicate significant differences at *p* < 0.05.

**Table 2:** Changes in whiteness of surimi gels with different concentrations of V_A_-WSE.

V_A_-WSE Concentration (%)	L*-Value	a*-Value	b*-Value	Whiteness
Control	79.17 ± 3.91 ^b^	−1.36 ± 0.28 ^b^	10.36 ± 0.33 ^ab^	76.64 ± 3.41 ^b^
2	81.57 ± 0.23 ^ab^	−1.48 ± 0.05 ^ab^	9.77 ± 0.09 ^cd^	79.08 ± 0.17 ^ab^
4	82.04 ± 0.18 ^ab^	−1.42 ± 0.14 ^ab^	9.62 ± 0.15 ^d^	79.57 ± 0.20 ^ab^
6	83.07 ± 0.78 ^a^	−1.57 ± 0.29 ^a^	10.16 ± 0.17 ^bc^	80.19 ± 0.77 ^a^
8	83.91 ± 0.05 ^a^	−1.42 ± 0.05 ^ab^	10.42 ± 0.17 ^ab^	80.78 ± 0.10 ^a^
10	83.73 ± 0.54 ^a^	−1.46 ± 0.16 ^ab^	10.71 ± 0.16 ^a^	80.46 ± 0.44 ^a^

V_A_-WSE: whey protein isolate–soybean lecithin-encapsulated vitamin A emulsion. The concentrations of V_A_-WSE in the prepared surimi gels were 0% (control), 2%, 4%, 6%, 8%, and 10%, respectively. Different lowercase letters (a–d) in the same column indicate significant differences at *p* < 0.05.

## Data Availability

The original contributions presented in this study are included in the article. Further inquiries can be directed to the corresponding author.
